# Bridging centrioles and PCM in proper space and time

**DOI:** 10.1042/EBC20180036

**Published:** 2018-11-14

**Authors:** Ramya Varadarajan, Nasser M. Rusan

**Affiliations:** Cell Biology and Physiology Center, National Heart, Lung and Blood Institute, National Institutes of Health, Bethesda, MD 20892, U.S.A.

**Keywords:** centrosomes, cellular reproduction, cell cycle

## Abstract

Throughout biology, specifying cellular events at the correct location and time is necessary for ensuring proper function. The formation of robust microtubule organizing centers (MTOCs) in mitosis is one such event that must be restricted in space to centrosomes to prevent ectopic MTOC formation elsewhere in the cell, a situation that can result in multipolar spindle formation and aneuploidy. The process of reaching maximum centrosome MTOC activity in late G2, known as centrosome maturation, ensures accurate timing of nuclear envelope breakdown and proper chromosome attachment. Although centrosome maturation has been recognized for over a century, the spatial and temporal regulatory mechanisms that direct MTOC activation are poorly understood. Here, we review Sas-4/CPAP, Asterless/Cep152, Spd-2/Cep192, and PLP/Pericentrin, a group of proteins we refer to as ‘bridge’ proteins that reside at the surface of centrioles, perfectly positioned to serve as the gatekeepers of proper centrosome maturation at the perfect place and time.

## Building centrosomes

**Figure 1:**
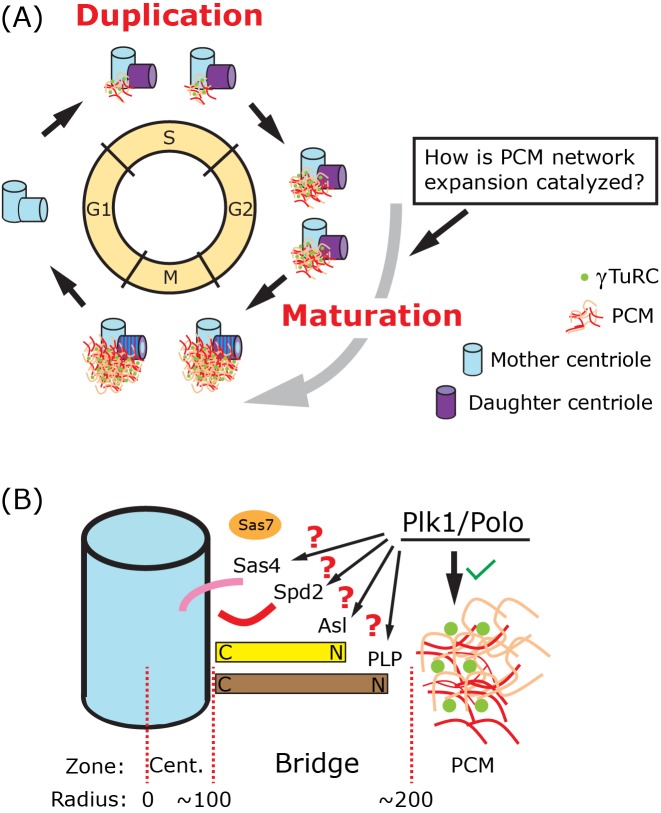
Bridge proteins facilitate centrosome maturation (**A**) Centrosomes undergo two critical cycles that are linked to the cell cycle. In S-phase, each mother centriole (blue) templates the nucleation of a new daughter centriole (purple), thereby forming two centrosomes. As cells near mitosis, centrosomes undergo maturation by recruiting additional PCM. The focus of this review is to explore the mechanism by which centrosome maturation is regulated (Box). How is PCM expansion catalyzed at the centrosome in late G2? (**B**) The three main centrosome zones are indicated: centriole, bridge, and PCM. The bridge zone is the area between the centriole (blue) and the PCM (colored network), spanning roughly the 100–200 nm position (radial distance). The bridge zone is occupied by four conserved proteins Sas-4/CPAP (pink), Spd-2/Cep192 (red), Asterless/Cep152 (yellow), and PLP/Pericentrin (brown). Spd-2/Cep192 also occupies the PCM zone as a critical member of the PCM network that also includes Cnn/CDK5RAP2 (orange) and γTuRCs (green). The *C. elegans* protein Sas-7 (orange oval) also qualifies as a bridge protein. Asterless/Cep152 and PLP/Pericentrin are radially organized such that their C-termini are anchored to the centrioles and N-termini are proximal to the PCM. Plk1/Polo is key to PCM network expansion, although substrates within the bridge zone have not been identified.

Centrosomes are non-membrane bound organelles composed of an orthogonal pair of centrioles surrounded by a protein network termed pericentriolar material (PCM). It is this PCM that nucleates and organizes microtubules (MTs) to form MT organizing centers (MTOCs). During early interphase (Figure 1A), a single centrosome radially organizes MTs used to traffic cargo, support cell shape, guide cell motility, and assist in cell signaling, among other roles. The centrosome then proceeds through two critical cycles [1]. The first is the Duplication Cycle, which is the process of going from one centrosome to two. The second is the Maturation Cycle, during which centrosomes anchor increasing amounts of PCM, nucleate more MTs, and become more robust MTOCs in preparation for mitosis where both centrosomes coordinate the assembly of the bipolar mitotic spindle (Figure 1A). Defects in centrosome protein function or in centrosome number cause many cellular abnormalities such as cell cycle arrest, aneuploidy, cell polarity defects, and missegregation of cell-fate determinants. Not surprisingly then, mutations in centrosome proteins are linked to many human diseases such as microcephaly and cancer [2–4].

A major advance in understanding these two cycles in recent years has been the positional mapping of centrosome proteins using structured illumination microscopy (SIM) [5–9]. Based on the distance from the center of the centriole, zones of centrosome proteins have been proposed. However, there is no consensus on the nomenclature of these zones, especially across species. In this review, we classify the centrosome as three hierarchical zones: (1) the centriole zone, (2) the ‘bridge’ zone, and (3) the PCM zone (Figure 1B). This review focuses on ‘bridge proteins’, which are ideally positioned between centrioles and PCM, and thus poised to facilitate the expansion of PCM during centrosome maturation. We propose that properly positioning and regulating bridge proteins is key to triggering centrosome maturation, which in-turn is critical for increased MTOC function, spindle formation, and ultimately chromosome segregation during mitosis.

## The centriole zone

Centrioles form the core structural unit of a centrosome. A relatively small number of centriole proteins are stereotypically organized and encased within a MT barrel with nine-fold symmetry. Studies over the past 10 years have made extensive progress toward our understanding of centriole assembly. Complex mechanisms involving Polo-like kinase 4 (Plk4 in humans and flies, Zyg1 in *Caenorhabditis elegans*) and the major centriole proteins Sas-4 (CPAP in human), Spd-2 (Cep192 in humans), Sas-6, Ana2/STIL/Sas5 (flies, human, *C. elegans*), and Cep135 ensure that a mother centriole templates the birth of a single daughter centriole once per cell cycle. These centriole duplication mechanisms have been recently reviewed [1,10]. Interestingly, clear evidence shows that centriole proteins, such as Spd-2 and Sas-4, also play roles in recruiting or assembling PCM, likely owing to their additional localization within the bridge and PCM zones. Investigating the dual roles for these proteins in centrosome duplication and maturation is challenging as complete loss-of-function analysis using null mutant or RNAi knockdown leads to loss of the entire organelle as a result of a failure in centriole duplication. Thus, a repeating theme throughout this review is the necessity for a deeper understanding of centriole proteins through structure-function analysis and separation-of-function mutations.

## The PCM zone, a network of Cnn–Spd-2–Pericentrin

The PCM is the outer layer of the centrosome and is composed of hundreds of proteins, including a matrix of Pericentrin (Pcnt/Kendrin in humans), Centrosomin (Cnn in flies, Cep215/CDK5RAP2 in humans), and Spd-2 (Cep192 in humans), which together function to recruit and anchor gamma tubulin ring complexes (γTuRC) [11–20]. *In vitro* work using *C. elegans* recombinant protein showed that Spd-5 (a protein many suggest to be a functional ortholog of Cnn/Cep215) and Spd-2 can form an expanding protein matrix, whose rate of assembly is enhanced by the critical mitotic kinase Polo-like kinase (Plk1 in human and *C. elegans*, Polo in flies) [21–24]. Because *C. elegans* do not have a Pcnt ortholog, the Spd-5/Spd-2 scaffold is likely the main PCM structure that recruits MT nucleating factors, be it γTuRCs or MT-associated proteins such as ZYG-9 and TPXL-1 [22].

In *Drosophila*, Polo is also critical for PCM assembly. One mechanism of Polo function is via phosphorylating the *p*hospho*re*gulated-*m*ultimerization (PReM) domain in the central region of Cnn [25], which then promotes either an intra- or inter-molecular interaction with the C-terminal CM2 domain of Cnn [26]. The *Drosophila* ortholog of Pcnt, Pericentrin-like protein (PLP), does not expand into the PCM zone and thus is not a component of the fly PCM network. However, as we will discuss below, PLP might serve as a catalyst to trigger the expansion and/or stabilization of PCM at the mother centriole.

In humans, the localization and function of Cep192 (Spd-2), Cep215/CDK5Rap2 (Cnn), and Pcnt appear to be significantly interdependent, but none rely exclusively on any one protein [20]. While less is known about mammalian PCM network formation, a mechanism similar to *Drosophila* and *C. elegans* is likely to emerge. In fact, in cultured mammalian cells it was shown that Plk1 phosphorylation of Pcnt is required for the recruitment of several PCM proteins, including Cep192 [27]; other Plk1 substrates have yet to be mapped.

Taking the work from various model systems in aggregate, a singular model of centrosome maturation is slowly coming into focus where Polo/Plk1 phosphorylates Cnn/Cep215/CDK5Rap2, Spd-2, and PLP/Pcnt to induce PCM expansion during G2. This expansion then greatly increases γTuRC recruitment and MT nucleation from the relatively low levels in interphase to the high levels needed for mitosis. A key question in the field remains – how is this network expanded or catalyzed at the right place (the surface of centrioles) and at the right time (in G2 during maturation) (Figure 1A, Box)?

## The bridge zone – templating the PCM network

We define ‘bridge’ proteins based on two criteria: the first is based on their position between the centriolar MT wall at roughly 100 nm (radial distance) and the inner edge of PCM at approximately 200 nm [6–9,28–30] (Figure 1B). This zone was referred to previously as ‘Zone II and III’ [9], the ‘inner and intermediate’ PCM [6], and in many studies simply as PCM. We use the term bridge as it is a descriptive term that conveys the function of these proteins to link centrioles and PCM. The second criterion for bridge proteins is the presence of published data to support their role in recruiting or anchoring PCM. It is important to keep in mind that these radial measurements vary between species and cell-type, and that centrosome protein positions are ‘normal distributions’, which means a significant amount of protein localizes on either side of the mean peak position. The significant overlap between the three centrosome zones probably explains the multifunctionality of centrosome proteins that the field is just beginning to appreciate.

Four scaffold proteins – Sas-4/CPAP, Spd-2/Cep192, Asl/Cep152, and PLP/Pcnt – fit the criteria of a bridge protein (Figure 1B). One additional *C. elegans* protein, Sas-7, is also properly positioned just beyond the centriole wall and has been implicated in PCM recruitment, thus satisfying the bridge protein criteria [31]. Interestingly, all of these proteins play an additional role in building or maintaining centrioles. This dual role has contributed to the significant challenge to independently investigate their PCM maturation role. We will discuss what is known about the role of bridge proteins in catalyzing and assembling the Spd-2–Cnn–Pcnt PCM network.

### Sas-4/CPAP

CPAP in humans, and Sas-4 in *C. elegans, Drosophila*, and mouse, is the most functionally diverse protein in the bridge category. Sas-4 is essential for centriole duplication in most organisms [18,32–34]. Earlier work showed that Sas-4 is, in fact, a centriole protein [35,36], while later SIM imaging specifically placed Sas-4 within the centriole zone of the newly forming daughter centriole [8,37]. This localization facilitates the role of Sas-4 in assembling the centriole MT wall [38] and in centriole length control [39,40]. Elegant work using a combination of structural and biochemical assays uncover how the two CPAP N-terminal MT-binding domains PN2.3 (LID + SAC regions) and MBD (MT binding domain) target CPAP to the plus end of centriole MTs. These two domains generate a perfect balance between promoting and restricting MT growth to ensure proper centriole length [41]. This work helps explain previous studies that showed that PN2.3 can suppress MT growth [42], while overexpression of CPAP can promote MT elongation [39,40,43]. In a separate role, several lines of evidence support a Sas-4 function in recruiting PCM. Work in *C. elegans* showed that partial depletion of Sas-4 reduces PCM levels [35], while work in human cell lines showed that CPAP immunoprecipitated with γ-tubulin, and that CPAP antibodies blocked MT nucleation from isolated centrosomes [44]. Additionally, SIM of cultured *Drosophila* S2 and human U2OS cells revealed Sas-4/CPAP localization as a ring around the mother centriole [8,37], within the bridge zone. We speculate that this mother centriole localization facilitates the secondary role of Sas-4 in recruiting PCM.

How might Sas-4/CPAP mediate PCM recruitment? There are currently two main models. The first model is direct tethering of PCM components to the centriole [45]. In this model, the C-terminal TCP domain of Sas-4 binds components of the centriole zone – STIL/Ana2 and Cep135 [46–49], while the N-terminus extends into the bridge zone to bind components of the PCM [37,50,51]. Consistent with this model, disruption of the TCP domain reduces PCM levels at centrosomes [45]. However, the mutation used in this study also led to centriole loss in 50% of cells, which would complicate analysis of PCM recruitment. Thus, the perfect separation-of-function mutation to independently test the role of Sas-4 in centrosome maturation remains elusive.

The second model of Sas-4 function involves a cell cycle regulated kinase signaling cascade [52]. Sas-4 is the only bridge protein to date shown to directly bind Polo [37], and work in *Drosophila* has recently shown that Cdk1/Cyclin B phosphorylation of Sas4-T200 is required for Polo recruitment to the centrosome [52]. Although not tested by Novak and colleagues, it is tempting to speculate that this Polo recruitment by Sas-4 occurs in G2 to initiate PCM network expansion by phosphorylating the Cnn–PReM domain at the surface of the centriole [53]. In support of this mechanism, Polo has been localized to the bridge zone [9] and the incorporation of PCM into the centrosome, at least in some cells, begins within the bridge zone (centriole wall) before expanding into the surrounding PCM zone [53,54].

For both the direct recruitment of PCM model [45] and the indirect signaling through Polo model [52], a stable pool of Sas-4 near the centriole wall is likely required. In fact, FRAP analysis of centrosomes has shown that the Sas-4 pool surrounding the mother centriole does not recover within the timeframe of centrosome maturation [53]. This does not exclude, however, a contribution from a dynamic pool of Sas-4 that might be recruited as part of a preassembled PCM complex such as the S-CAP complex shown in *Drosophila* [51].

Taken together, Sas-4 is a complicated protein with multiple roles and many levels of regulation. Similar to all the bridge proteins, separation of function mutation analysis will be required to independently interrogate Sas-4’s roles in centriole duplication, centriole elongation, and PCM recruitment.

### Asterless/Cep152

Asl and its human ortholog Cep152, much like Sas-4/CPAP, possess dual functions in centriole duplication and centrosome maturation. Asl/Cep152 is critical for centriole duplication, as it functions to recruit the centriole duplication kinase Plk4 to the centriole [55–62]. Asl displays a highly ordered molecular architecture within the bridge zone [5–9] with its C-terminus close to the centriole wall, anchored and properly positioned by Ana1/Cep295 [5], Cep135 [37], and possibly Sas-4 [57,60,62]. The N-terminus of Asl extends radially away from the centriole toward the PCM zone and is thus well positioned to influence PCM assembly (Figure 1B). In fact, several studies in *Drosophila* have shown that loss of Asl leads to a reduction in PCM levels [53,55,63]. In mammalian cells, depletion of Cep152 resulted in a reduction of γ-tubulin, although the precise cell cycle stage was not reported [64], and therefore it is not clear if Cep152 is required for centrosome maturation per se.

Interestingly, Asl is loaded onto daughter centrioles between metaphase and telophase [55] as part of the ‘centriole-to-centrosome conversion’ mechanism that transforms an immature daughter centriole into a mother centriole competent to duplicate and recruit PCM [5,52]. To date, however, nothing is known about the regulation of Asl for the precise timing of PCM recruitment much later in the cell cycle in G2. Is Asl/Cep152, for example, phosphorylated by Polo or another mitotic kinase in G2 (Figure 1B)?

Likewise, little is known about the events downstream of Asl – how exactly does Asl feed into templating or anchoring the Cnn–Spd-2 PCM network during maturation? We speculate that this is mediated through direct binding and recruitment of Spd-2 and/or Cnn to the surface of the centriole as extensive direct interactions have been reported among all three proteins in *Drosophila* [25,29], and proximity labeling experiments suggest Cep152–CDK5Rap2 interact in mammalian cells [64]. These direct interactions might serve to concentrate Spd-2 and Cnn at the centriole wall and bring them in contact with Polo kinase for activation.

An important note regarding these aforementioned *Drosophila* studies on Asl PCM recruitment is that they were either conducted using a poorly characterized allele of Asl (*asl^1^*) [55,63] which was later shown to be hypomorphic [56], or in *Drosophila* embryos using inhibitory antibody injections [65]. Furthermore, in contrast with these studies, two studies using the null allele *asl^mecD^* in *Drosophila* have shown that Asl-free centrioles are fully capable of recruiting PCM in male meiosis [29,56]. Thus, extensive future structure function studies in mammalian and *Drosophila* systems (*C. elegans* do not have Asl) are required to understand how Asl functions to regulate PCM recruitment independent of its role in centriole duplication.

### Spd-2/Cep192

We have included Spd-2 in the list of bridge proteins because it satisfies both bridge protein criteria of localization and a known role in PCM recruitment. Spd-2, however, is a major component of the PCM itself as we describe above and is critical for centriole duplication in several species [18,21,59,66,67]. The current published data, however, does attribute a distinct role for Spd-2 in the bridge zone in catalyzing the initial steps of centrosome maturation, and thus we do not further discuss Spd-2 in this review. However, we do postulate that protein modification of bridge zone Spd-2 (at the centriole surface) is an important trigger of PCM expansion.

### Pericentrin/PLP

Pericentrin (Pcnt) has an extensive research history, most of which describes Pcnt as a critical PCM protein that is incorporated throughout the expanding PCM in mitosis, similar to Spd-2/Cep192, Cnn and γ-tubulin. However, there is a pool of Pcnt in the bridge zone as identified by SIM of interphase cells [6,8]. How then might we investigate the bridge role of Pcnt independently of its role within the PCM? In this respect, the *Drosophila* ortholog of Pcnt, PLP (CP309) has proven to be quite valuable. In *Drosophila*, PLP does not assemble with the Cnn–Spd-2 network in mitosis but remains tightly associated with the centriole, similar to its localization in interphase. Thus, studying PLP affords an opportunity to independently investigate the bridge role of Pcnt/PLP without the confounding function within the PCM.

In *Drosophila*, loss of PLP results in the reduction and disorganization of Cnn in mitosis [68,69], suggesting that PLP at the centriole wall does, in fact, influence the recruitment and stability of the Spd-2–Cnn network. The one exception to this strict PLP bridge zone localization is seen in *Drosophila* embryos where PLP-satellites are found in the interphase centrosome ‘flare zone’, which extend over 1.5 μm in radius, well beyond the PCM zone [28]. Because the flare zone appears to be a *Drosophila* embryos specific structure, other *Drosophila* cell types are more suitable for studying the bridge role of PLP.

Similar to the molecular architecture of Asl, the C-terminus of PLP (and Pcnt) is anchored to the centriole, while the N-terminus radially extends toward the PCM zone [6–9]. This organization is consistent with the extended molecular characteristic of Spc110, the yeast ortholog of Pcnt, within the yeast spindle pole body [70,71], suggesting an evolutionarily conserved molecular architecture. Additional structural insight into PLP/Pcnt was gained using STORM imaging and EM, which revealed symmetric clusters around the centrioles, reminiscent of the nine-fold symmetry of the centriolar MTs [7,8]. This radially organized and extended conformation of PLP might directly account for its role in PCM recruitment and organization, but this hypothesis has yet to be tested.

Another similarity shared by Asl and PLP is the timing of PLP loading onto the centriole. PLP appears to load onto the daughter centriole just after Asl during centriole-to-centrosome conversion in metaphase/anaphase [5]. Thus, the addition of both PLP and Asl to daughter centrioles might be required for fully converting a centriole into a mature organelle competent for recruiting PCM much later in G2. But again, few details are known about the regulation of PLP. Based on mammalian work where Plk1 phosphorylates Pcnt to allow for PCM recruitment [27], a reasonable hypothesis is that PLP is also a Polo substrate that can then trigger, or catalyze Spd-2–Cnn network expansion through direct protein–protein interactions. In fact, extensive direct PLP–Cnn and PLP–Spd-2 interactions have been reported [28,37,72,73]. Interestingly, many interactions overlap with one another. For example, the CM2 domain of Cnn can interact with the central region of Cnn [26,37] and the N-terminal region of PLP [28,37]. These types of overlapping interactions drive hypotheses of competitive and cooperative protein binding that is potentially regulated by the biochemical state in different cell cycle stage. At this point, however, such hypotheses lack experimental support.

## Concluding remarks

In this review, we discuss the architecture of the centrosome in the terms of three zones, focusing mainly on the central zone of the centrosome we refer to as the ‘bridge zone’, which comprises four centrosome proteins (Sas4/CPAP, Asl/Cep152, Spd-2/Cep192, and PLP/Pcnt). One additional protein that we do not discuss in this review is *C. elegans* Sas-7, which also likely functions as a bridge protein [31]. These bridge proteins reside just outside of the centriole wall and have been shown to play an important role in PCM assembly, likely through parallel mechanisms that cross-communicate. Perturbation of any one mechanism does not completely abolish centrosome function, possibly due to system redundancy that ensures robust MTOC activity needed for spindle formation. Polo is clearly a critical component of centrosome maturation and we predict that future work will identify all four bridge proteins as Polo/Plk1 substrates. Determining the impact of phosphorylation (by Polo or other mitotic kinases) on the centrosome interaction network, such as those shown for Sas-6 [1], Cep135 [37], and Cnn [26], will be quite exciting. Identifying these sites and their function will constitute an important advance in our understanding of how centrosome maturation and MTOC activity is properly triggered in G2. Furthermore, showing that bridge proteins are, in fact, the key proteins in templating PCM network expansion will help explain how MTOC activity is spatially restricted to centrosomes. In addition to the clear need for generating separation-of-function mutations, understanding these spatial and temporal mechanisms will be aided by advancements in optogenetics and rapid protein degradation systems such as the auxin-induced degradation (AID) systems, which provide a way to carefully manipulate protein dynamics in space and time.

## Summary

- Centrosomes undergo a dramatic transformation in G2, known as centrosome maturation, where additional PCM is recruited. This maturation facilitates an increase in MT nucleation and organization in preparation for mitosis.
- The region of the centrosome just beyond the centriole wall is a zone critical for centrosome maturation: we term this region as the ‘bridge zone’.
- This zone is occupied by four proteins referred to as ‘bridge proteins’, all of which have been shown to play a role in some aspect of centrosome maturation. The bridge proteins are Sas-4/CPAP, Asterless/Cep152, Spd-2, and PLP/Pericentrin. The *C. elegans* specific protein Sas-7 is also classified as a bridge protein.
- Polo/Plk1 is an essential kinase for centrosome maturation and has a role in regulating bridge proteins. It is predicted that future work will identify all bridge proteins as Polo/Plk1 substrates and that their phosphorylation is required to catalyze PCM expansion in G2.

## Abbreviations

MT: microtubule
MTOC: microtubule organizing center
PCM: pericentriolar material
Plk4: Polo-like kinase 4
PLP: Pericentrin-like protein
PReM: phosphoregulated-multimerization
SIM: structured illumination microscopy
γTuRC: gamma tubulin ring complex
